# Impact of orthognathic surgery on quality of life in patients with different dentofacial deformities: longitudinal study of the Oral Health Impact Profile (OHIP-14) with at least 1 year of follow-up

**DOI:** 10.1007/s10006-021-00992-6

**Published:** 2021-07-29

**Authors:** Jacco G. Tuk, Jerome A. Lindeboom, Misha L. Tan, J. de Lange

**Affiliations:** 1 Department of Oral and Maxillofacial Surgery, Amsterdam UMC, University of Amsterdam, Amsterdam, Netherlands; 2 Amstelland Hospital Amstelveen, Meibergdreef 9, 1105 AZ Amsterdam, Netherlands

**Keywords:** Orthognathic surgery, Pain, OHRQoL

## Abstract

**Objective:**

The objective of this study was to assess the impact of orthognathic surgery for dental facial deformities on oral health-related quality of life (OHRQoL) in the immediate postoperative period up to at least 1 year after surgery.

**Study design:**

This prospective study evaluated data from 85 patients. OHRQoL was assessed using the Dutch version of the Oral Health Impact Profile questionnaire (OHIP-14NL) preoperatively (T_0_), each day for 7 days postoperatively (T_1_–T_7_) and 4 weeks (T_8_), 6 months (T_9_), and at least 1 year (T_10_) after surgery. The total OHIP score was calculated for each patient, with higher OHIP scores indicating a worse impact on oral health. Patients also completed an extra questionnaire about self-care, discomfort, and experienced pain (rated on a 10-point scale) in the postoperative period (T_1_–T_10_).

**Results:**

The mean OHIP score increased sharply at T_1_ compared to T_0_ but decreased significantly in the first postoperative week. The mean OHIP score at T_8_ was still higher than before surgery. However, at T_9_ and T_10_, the mean OHIP score was significantly lower than at T_0_ (*P* < .05). No significant difference in OHIP score was found between gender, age, type of surgery, and indication for surgery. Pain significantly decreased from T_6_ to T_0_. The OHIP and pain scores significantly positively correlated at every time point except T_9_.

**Conclusion:**

The findings indicate that OHRQoL is reduced from baseline in the immediate postoperative period but improves over time. By 1 year, OHRQoL improves significantly after orthognathic surgery in patients with dentofacial deformities.

## Introduction

Many studies have found lower oral health-related quality of life (OHRQoL) in patients with dentofacial deformities [1–7]. Patients with dentofacial deformities are characterized by various irregularities of the face and dental bone structures, such as hyperplasia, hypoplasia, and asymmetries of the maxilla, mandible, or chin. An abnormal position of the jaws can manifest in the dentition as a class II or III malocclusion and cause esthetic and functional problems, including difficulty chewing, sleeping, breathing, speaking, or overall oral health problems [8]. Some patients experience psychological and emotional problems [9].

Orthognathic surgery is a common treatment for dentofacial deformities. The procedure involves repositioning of the maxilla, mandible, or both, sometimes in combination with correction of the chin. The functional and esthetic goals are to achieve a class I dental occlusion and facial balance and proportion. Traditionally, orthognathic surgery involves preoperative and postoperative orthodontics to achieve dentofacial correction by aligning the dental arches. The main surgical techniques are Le Fort I osteotomy, bilateral sagittal split osteotomy (BSSO), and bimaxillary osteotomy (BIMAX), which are sometimes combined with an osseous genioplasty.

Patients seek orthognathic surgery for various reasons. Their primary motivations are esthetic concerns and improved QoL [10, 11]. Some studies have found that oral function, including bite, pain, smile, and speech, is a primary motivation [12–14]. A recent systematic review showed physiological and psychological improvement in QoL following orthognathic surgery [15]. A study with a 5-year follow-up found significant improvement and stabilization after 2–5 years in regard to the general health-related QoL, OHRQoL, and psychosocial function after BSSO [16].

The Oral Health Impact Profile (OHIP-14) is a standardized questionnaire that measures the OHRQoL. The questionnaire is a short version of the OHIP-49 that includes 14 questions representing 7 domains [17, 18]. The Dutch version of the questionnaire, OHIP-14NL, was reported in 2011 to be a reliable and valid questionnaire for measuring the impact of oral health on QoL [19]. Other validated questionnaires commonly used in orthognathic studies are the Orthognathic Quality of Life Questionnaire (OQLQ) and the Short Form Health Survey (SF-36) [15].

It is important to provide patients with realistic and accurate information prior to the start of orthognathic treatment. The temporary discomfort in the initial postoperative period, such as problems related to oral function, pain, numbness of the lower lip and chin, and postoperative bleeding and swelling, should be explained to patients prior to the treatment, and they should also be given a realistic idea of the final facial appearance [20–22]. This knowledge would lead to greater satisfaction after surgery [12, 23, 24].

The aim of this study was to evaluate the impact of orthognathic surgery on the QoL of patients with various dentofacial deformities in the immediate postoperative period and during at least 1 year of follow-up using the OHIP-14 questionnaire. The hypothesis is that the QoL of patients with different dentofacial deformities improves with orthognathic surgery. This knowledge would be useful in improving preoperative, perioperative, and postoperative care and could lead to greater satisfaction for patients.

## Materials and methods

### Study design and ethical approval

This prospective observational study was approved by the medical ethics committee (METC W17_083#17.102) of Amsterdam University Medical Center (Amsterdam UMC, location AMC). It was granted a non-WMO status (Medical Research Involving Human Act).

### Patients

Patients were eligible for the study when they had facial skeletal malformations that required elective combined treatment with preoperative and postoperative orthodontic corrections and orthognathic surgery at Amsterdam UMC, location AMC, between September 2016 and March 2020. The patients were selected for the study by an oral maxillofacial surgeon. The inclusion criteria were age ≥ 18 years; ASA class 1, 2, or 3; no congenital anomalies, including cleft lip and/or palate; and sufficient command of the Dutch language. Exclusion criteria were obstructive sleep apnea syndrome as the reason for treatment, craniofacial syndromes, and previous history of orthognathic surgery. All participants were informed about the aims and protocol of the study and provided informed consent.

### Planning and surgery

Each patient underwent preoperative orthodontic alignment with fixed orthodontic braces for approximately 18 months. They also received postoperative orthodontic alignment with fixed orthodontic braces for another 6 months. Analysis and treatment planning were carried out with study models mounted on an adjustable articulator to facilitate three-dimensional planning and manufacturing of the interocclusal positioning wafers. Patients in the study received one of the following surgical corrections: Le Fort 1 osteotomy, bilateral sagittal split osteotomy (BSSO), bimaxillary osteotomy (BIMAX), or osteotomy combined with genioplasty. Postsurgical stabilization was achieved with elastics during the first 2 weeks of healing. The patients were followed up for at least 1 year after surgery.

### Data collection

Demographic information (gender, date of birth) and information about the surgery (date of surgery, type of surgery, indication for surgery, blood loss, and time of surgery) were collected from the medical records for each patient included in the study. The patients were asked to complete a questionnaire before the operation (T_0_, baseline) and every day for the first 7 days after the surgery (T_1_–T_7_). The next questionnaires were completed postoperatively at 4 weeks (T_8_), 6 months (T_9_), and at least 1 year (T_10_). During the first 6 months of the study, the patients received a written questionnaire; thereafter, online questionnaires were sent by email. As a result, some patients received all of the questionnaires online, whereas others received only the last one online. LimeSurvey 2.6.4. was used as a tool for online surveys and quota management (LimeSurvey GmbH, LimeSurvey (2.6.4.) https://limesurvey.amc.nl/). Patients received two reminders if they did not respond after 1 week. The questionnaire used for this study was OHIP-14NL [19]. This questionnaire focuses on the impact of a person’s oral health on QoL, evaluating the following domains: functional limitation, physical pain, psychological discomfort, physical disability, psychological disability, social disability, and handicap. Answers to each question indicated the frequency of occurrence, with five possible answers: 0 = never, 1 = hardly ever, 2 = occasionally, 3 = fairly often, and 4 = very often. The total OHIP score was the sum of the answers to the 14 questions. Scores ranged from 0 to 56, with higher scores indicating a worse impact on oral health. In addition to the OHIP-14 questions, patients responded to four relevant questions that covered pain experienced (rated on a 10-point scale), self-care applied, discomfort experienced, and the use of pain medications.

### Statistical analysis

The Shapiro–Wilk test was applied to verify the data distribution and normality. The data were not normally distributed, so nonparametric tests were used. Friedman two-way analysis of variance and a post hoc test were performed to investigate the change from baseline over 1 to 7 days after surgery. The Wilcoxon signed rank test was used to investigate the change between two time points (4 weeks (T_8_), 6 months (T_9_), or 1 year (T_10_) compared to baseline and the change per OHIP question between baseline (T_0_) and at least 1 year (T_10_). Correlations were analyzed by the Spearman rank correlation coefficient. The Kruskal–Wallis test was used to analyze the difference between the OHIP score and type of surgery or indication for surgery. A *P* value < 0.05 was considered significant. SPSS Statistics (version 26.0 IBM Inc., Armonk, NY) for Mac was used for statistical analyses.

## Results

### Demographic data

A total of 94 patients were included in the study. Nine patients were excluded during the study because they did not respond to any of the questionnaires. The final data were based on answers from 85 patients (48 females and 37 males). The patient characteristics are given in Table 1. No difference was found between men and women in regard to age, type of surgery, indication for surgery, blood loss, or duration of surgery. Blood loss correlated with the duration of surgery (*r* = 0.542, *P* < 0.000, *n* = 83), with more blood loss occurring with a longer time in surgery. All data were anonymized.

**Table 1:** Demographic data

	Total	Female	Male	*P* value
Patients	85 (100)	48 (56.5)	37 (43.5)	
Age, years				.496
Range	28.6 ± 10.6 18–60	27.9 ± 10.7	29.5 ± 10.5	
Type of surgery				.139
Le Fort I Osteotomy	15 (17.6)	8 (16.7)	7 (18.9)	
BSSO	33 (38.8)	21 (43.8)	12 (32.4)	
BIMAX	24 (28.2)	15 (31.3)	9 (24.3)	
Osteotomy with genioplasty	13 (15.3)	4 (8.3)	9 (24.3)	
Indication for surgery				.132
Class II	55 (64.7)	34 (70.1)	21 (56.8)	
Class III	29 (34.1)	14 (29.1)	15 (40.5)	
Class I (anterior open bite)	1 (1.2)	0	1 (2.7)	
Blood loss, mL	275.5 ± 240.7	247.2 ± 244.4	312.5 ± 234.25	.223
Duration of surgery, min	151.8 ± 66.3	145.8 ± 66.3	159.7 ± 66.3	.341

Data are given as n (%) or mean ± standard deviation unless otherwise noted

*n*, number; *min*, minutes significance at P < .05

### OHIP scores

Table 2 shows the mean OHIP score measured over all time points. Higher OHIP scores indicate lower OHRQoL. The range of T_10_ is 1–3 years. A correlation was found between the duration of surgery and the OHIP score on the first 7 days after surgery (*r* = 0.3 − 0.4, *P* < 0.05). The longer the surgery, the higher the OHIP score in the first week. No significant correlation was found between age, gender, blood loss, or OHIP score.

**Table 2:** Mean OHIP score (SD) over all time points

Time point	Mean ± *SD*	*n*
Preoperative (T_0_)	15.3 ± 10.4	76
Day 1 (T_1_)	32.4 ± 12.5	63
Day 2 (T_2_)	33.3 ± 13.3	58
Day 3 (T_3_)	33.6 ± 12.9	60
Day 4 (T_4_)	31.2 ± 12.5	59
Day 5 (T_5_)	32.1 ± 12.0	59
Day 6 (T_6_)	30.0 ± 12.7	60
Day 7 (T_7_)	27.8 ± 13.3	57
4 weeks (T_8_)	22.2 ± 12.5	69
6 months (T_9_)	9.2 ± 7.8	46
≥ 1 year (T_10_)	7.2 ± 7.8	40

*SD*, standard deviation

The Friedman test was used to assess the mean OHIP scores over baseline (T_0_) and the first 7 days after surgery (T_1_–T_7_). The overall *P* value < 0.00 indicated a significant overall difference in mean OHIP scores between the first 7 days. The mean OHIP score increased from T_0_ to T_1_ but tended to decrease from T_1_ to T_7_. A post hoc pairwise comparison indicated that the OHIP score from T_1_ to T_6_ was significantly higher than at T_0_, but there was no significant difference between T_0_ and T_7_ (Table 3).

**Table 3:** Mean difference in OHIP score in the first week compared to baseline (*n* = 49)

	Mean difference	*P* value
T_0_–T_1_	17.1	.000
T_0_–T_2_	18.0	.000
T_0_–T_3_	18.3	.000
T_0_–T_4_	15.9	.000
T_0_–T_5_	16.8	.000
T_0_–T_6_	14.7	.011
T_0_–T_7_	12.5	.279

Significant at *P* < .05

According to the Wilcoxon signed rank test, the mean OHIP score was still higher 4 weeks after surgery compared to baseline (*P* = 0.002) but lower at 6 months and 1 year compared to baseline (*P* = 0.000; Table 4).

**Table 4:** OHIP score at 4 weeks, 6 months, and 1 year compared to baseline

	Mean difference	*P* value	*n*
Baseline (T_0_), 4 weeks (T_8_)	6.9	.002	58
Baseline (T_0_), 6 months (T_9_)	− 6.1	.000	46
Baseline (T_0_), at least 1 year (T_10_)	− 8.1	.000	38

*n*, number

Significant at* P* < .05

The statistical analysis of the changes in the total OHIP score over time indicated that the OHRQoL decreased sharply immediately 1 day after surgery and then improved slowly but was still lower than the baseline at 4 weeks. At 6 months, the OHRQoL was better than before surgery and continued to improve for at least 1 year. When the change in mean OHIP-14 score was examined per question after at least 1 year (T_10_) compared to baseline (T_0_), the Wilcoxon signed rank test showed a significant reduction for all questions except question 14 (Table 5).

**Table 5:** OHIP score per question: at least 1 year after surgery compared to baseline

Question in Table 1	Problem	Mean T_0_ (*SD)*	Mean T_10_ (*SD*)	Mean difference (*SD*)	*P* value
1	Pronunciation	1.1 (1.1)	0.6 (0.8)	0.7 (1.0)	.001
2	Reduced taste	0.4 (0.7)	0.1 (0.3)	0.3 (0.9)	.015
3	Painful aching	1.3 (1.3)	1.0 (1.0)	0.6 (1.4)	.026
4	Discomfort in eating	1.5 (1.4)	0.7 (1.0)	0.5 (1.7)	.000
5	Self-consciousness	1.8 (1.2)	0.9 (1.1)	1.3 (1.4)	.000
6	Feeling tense	1.5 (1.1)	0.7 (1.0)	1.0 (1.2)	.000
7	Unsatisfactory diet	0,9 (1.1)	0.4 (0.9)	0.3 (1.9)	.001
8	Interruption of meals	0,6 (0,9)	0.3 (0.6)	0.3 (1.0)	.012
9	Difficulty relaxing	1.0 (1.2)	0.7 (0.9)	1.9 (1.2)	.013
10	Embarrassment	2.0 (1.2)	0.5 (1.1)	1.8 (1.4)	.000
11	Irritability	0,7 (0,9)	0.4 (0.7)	1.3 (1.2)	.002
12	Difficulty with normal tasks	0.7 (1.0)	0.3 (0.7)	0.6 (1.1)	.000
13	Life less satisfying	1.2 (1.1)	0.5 (0.7)	1.0 (1.1)	.000
14	Totally unable to function	0.4 (0.7)	0.2 (0.5)	0.2 (0.1)	.091

*SD*, standard deviation

Significance at *P* < .05

Question 14, which concerns inability to function, had a mean score of 0.4 at the baseline. This low score indicated that patients did not or hardly ever experienced problems with function before surgery.

The Kruskal–Wallis test was used to assess the difference in the mean OHIP score across the different types of surgery and indications for surgery at a single time point (*P* > 0.05). We found no significant difference in the mean OHIP score between the four types of surgeries (Table 6) and between the two indications for surgery (class II vs. class III; Table 7) at any single time point. Class I with anterior open bite was not analyzed because only one patient had this deformity.

**Table 6:** OHIP scores and type of surgery over time (± SD)

	Le Fort I osteotomy	BSSO	BIMAX	Osteotomy with genioplasty	*P* value	*N*
Baseline (T_0_)	14.5 ± 8.6	17.7 ± 11.8	18. 8 ± 12.4	8.7 ± 6.0	.073	76
4 weeks (T_8_)	20.1 ± 11.7	21.4 ± 11. 8	26.1 ± 14.1	20.9 ± 12.4	.612	59
6 months (T_9_)	9.1 ± 7.0	11.6 ± 8.7	8.9 ± 8.6	7.3 ± 7.1	.612	46
1 year (T_10_)	6.5 ± 7.7	8.6 ± 10.6	8.1 ± 8.0	7.2 ± 7.8	.941	40

*SD*, standard deviation; *N*, number of patients

Significance at *P* < .05

**Table 7:** OHIP scores and indication for surgery over time (± SD)

	Class II	Class III	*P* value	*N*
Baseline (T_0_)	14.2 ± 10.3	17.6 ± 10.3	.161	76
4 weeks (T_8_)	23.5 ± 13.0	19.9 ± 11.5	.388	59
6 months (T_9_)	9.2 ± 7.5	9.1 ± 8.6	.905	46
1 year (T_10_)	7.0 ± 7.8	7.7 ± 7.9	.766	40

*SD*, standard deviation; significance at *P* < *.05*; *N*, number of patients

Two-way ANOVA test was used to assess the average difference between the types of surgeries and types of deformities across four time points. No significant difference was found in the mean OHIP scores between the different types of surgeries over time (*P* = 0.783) and all time points together (*P* = 0.305). There was also no significant difference between class II and class III patients over time (*P* = 0.905) and all time points together (*P* = 0.860).

### Pain score

**Fig. 1:**
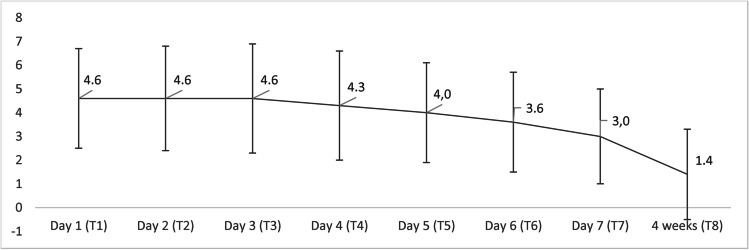
Mean pain score over time (*n* = 46). Error bars indicate standard deviation

The pain score was measured on a scale from 0 to 10 from day 1 (T_1_) to at least 1 year (T_10_) after the operation. Because of the low response rate to the questionnaire, it was analyzed from day 1 to week 4 (Fig. 1). The Friedman test was used to analyze the data, showing a significant decrease in the mean pain score from day 6 compared to day 1 (*n* = 46). Pain scores significantly positively correlated with OHIP scores for every time point except for 6 months (T_9_) (Table 8). No correlation was found between pain and age, gender, blood loss, time of surgery, indication for surgery, or type of surgery (*P* > 0.05).

**Table 8:** Correlation between OHIP score and pain score for all time points

Time point	*n*	*R*	*P* value
Day 1 (T_1_)	63	0.528	.000
Day 2 (T_2_)	58	0.484	.000
Day 3 (T_3_)	59	0.424	.001
Day 4 (T_4_)	58	0.394	.002
Day 5 (T_5_)	58	0.427	.001
Day 6 (T_6_)	59	0.312	.016
Day 7 (T_7_)	56	0.522	.000
4 weeks (T_8_)	59	0.494	.000
6 months (T_9_)	46	0.135	.371
≥ 1 year (T_10_)	40	0.315	.048

*n*, number; *R*, correlation coefficient

Significance at* P* < .05

### Additional questions about self-care and discomfort

The additional questions about self-care, pain, and discomfort were filled in by the patients for time points T_1_–T_10_ using “yes” or “no” (Table 9). Chi-squared indicated no significant difference between men and women. The need for self-care and presence of discomfort were high in the immediate postoperative period. More than 50% of the patients needed pain medication for the first 7 days and cooling with ice for the first 3 days. After 4 weeks, more patients were able to do without any extra self-care measures (men, 74%; women, 73%). In addition, a high percentage of patients experienced discomfort in the immediate postoperative period. More than 50% of the patients felt some discomfort 6 months after the surgery. After 1 year, limitations in mouth opening, swelling of the cheeks, and pain resulting from the surgery were absent in almost all patients, though some patients experienced other discomfort.

**Table 9:** Percentage of patients answering self-care and discomfort questions after surgery

Question		Day 1	Day 2	Day 3	Day 4	Day 5	Day 6	Day 7	4 weeks	6 months	≥ 1 year
	Male (*n*) Female (*n*)	28 35	26 32	25 34	25 33	26 32	25 34	24 32	27 33	20 27	18 23
Did you use any pain medication?	Male Female	100 92	89 91	88 94	84 91	89 85	84 86	79 70	27 24	6 0	0 4
Did you cool with an ice pack?	Male Female	71 74	50 56	48 53	32 61	27 38	32 12	21 9	4 0	0 4	0 4
No extra self-care was needed	Male Female	0 3	8 6	12 6	16 6	12 9	16 18	25 31	74 73	95 96	89 96
Did you experience limited mouth opening?	Male Female	96 94	100 100	100 88	96 94	100 91	92 91	96 88	67 70	25 22	0 13
Did you experience reduced chewing ability?	Male Female	93 83	96 94	88 94	92 97	96 87	92 91	96 88	85 79	20 19	0 4
Did you have a swollen cheek?	Male Female	89 97	100 100	96 97	96 97	96 94	96 85	96 81	48 39	5 4	0 4
Did you have pain as a result of surgery?	Male Female	82 69	85 75	76 76	76 70	77 69	68 59	58 56	22 18	5 7	6 4
Did not experience any discomfort	Male Female	4 3	0 0	0 0	0 0	0 0	4 0	4 0	7 9	45 48	50 52
Other discomfort	Male Female	4 17	15 16	12 9	12 9	8 9	8 12	8 13	11 12	15 30	50 34

## Discussion

The aim of this study was to investigate the impact of orthognathic surgery on OHRQoL in the immediate postoperative period until at least 1 year, as measured by the OHIP-14NL questionnaire. The OQLQ is another commonly used questionnaire in orthognathic studies. A comparison of the OQLQ with the OHIP-14NL has shown that both tools are able to discriminate differences in QoL over time and between patient groups. The OQLQ is more specific for orthognathic surgery [25]. The English version of the OQLQ was developed in 2000 and validated in 2002 [6, 25–27]. However, the current study did not use the OQLQ because the Dutch version has not yet been validated. The SF-36 is also used in some orthognathic studies, but it focuses more on one’s physical and mental status [25]. The SF-36 was not used in this study because this questionnaire is not restricted to the orofacial area.

Previous studies have reported a lower QoL in patients with dental facial deformities compared to a control group [28–30]. The present study did not have a control group. The preoperative OHIP score in this study was higher than the OHIP scores of control groups in other studies. Thus, in general, one can conclude that the OHRQoL of persons with dentofacial deformities is worse overall than in patients without a dentofacial deformity.

The current study found significant deterioration of the OHRQoL 1 day after surgery compared to baseline. However, the OHRQoL improved significantly in the first week. The OHRQoL was still significantly lower after 4 weeks but after 6 months had improved. Comparable results after orthognathic surgery have been reported in other studies [8, 27–33]. Deterioration in the immediate postoperative period has also been described in patients who suffer from pain, swelling, limited mouth opening, reduced masticatory efficiency, and numbness of the lower lip [28, 34, 35]. The answers to the additional questions in our study indicate that a high proportion of patients experience discomfort and need more self-care in the immediate postoperative period. This study also found a significant positive correlation between duration of surgery and OHIP score for the first 7 days after surgery. There was no significant correlation between OHIP score and age or gender.

Some studies have described female patients experiencing better improvement in self-esteem and a greater reduction in depression after orthognathic surgery compared to male patients [3, 28, 29]. Corso et al. found, in both the dentofacial deformities group and control group, a lower perception of QoL by women compared to men [28]. However, some studies did not find a difference in OHIP score between men and women [31, 32, 34]. The present study also found no difference in OHIP score between men and women.

This study found no difference in regard to the type of surgery. However, some investigators have found better improvement in patients who underwent BIMAX compared to single jaw surgery (Le Fort I or BSSO) [29]. Another study evaluated whether a combination of BIMAX and genioplasty for females with prognathism and maxillary hypoplasia has a greater positive impact on QoL than BIMAX alone; genioplasty led to significantly greater QoL after surgery [36].

The current study did not find a significant difference between indications for surgery. Some other studies also found no significant association between the indication for surgery and OHIP-14 scores [28, 34]. However, other studies have found that skeletal class III patients had more positive effects form surgery than class I and class II patients [29, 32]. Baherimoghaddam et al. found an improvement in both class II and class III patients, but the pattern of change was different; class II patients experience deterioration in QoL during the preoperative stage and improvement in function rather late in the postoperative stage [8]. Class III patients exhibited more significant changes in the domains concerning appearance and psychological issues.

Another finding in this study was that the OHIP score for every question was significantly lower at least 1 year after the operation compared to baseline, except for question 14, which refers to total oral dysfunction. The fact that the OHIP score for question 14 was only 0.4 at baseline indicates that people with various dentofacial deformities do not or hardly suffer from total oral dysfunction. This could explain why no improvement was noted after 1 year. The patients recruited for this study may have more problems with their facial appearance psychologically than with function.

The pain score significantly decreased after day 5 and was very low after 4 weeks. In the first week, a high percentage of patients said that they had taken painkillers. This could influence the perceived pain, so the actual pain score may have been higher. There was a significant positive correlation between pain scores and OHIP scores for every time point except 6 months, but no association was found between pain and age, gender, blood loss, time of surgery, indication for surgery, or type of surgery.

A major limitation of this study is that only 22 of the 85 patients completed all the questionnaires. A paper version of the questionnaires was used only in the first 6 months of this study. After that, the questionnaire was sent by email; patients may have perceived the questionnaires received by email as less important, despite the reminders that were sent. Consequently, the number of patients was too low for all 11 time points (T_0_–T_10_). Therefore, we applied the Friedman test for only the first 7 days after surgery and separately tested the later time points using the Wilcoxon signed rank test.

In this study, some patients mentioned numbness of the lower lip in the comments to the questionnaire, though numbness of the lower lip after surgery was not specifically requested. There may have been more patients who suffered from this complication. Damage of the inferior alveolar nerve is a common postoperative complication [34–36]. There is broad variation in the incidence of inferior alveolar nerve injury [37, 38], which could influence patient satisfaction [39]. However, some studies that report a high incidence of lip paresthesia in patients following orthognathic surgery have shown no effect on patient satisfaction [9, 40, 41]. Most patients, especially in the younger age group, seem to adapt to this complication [40].

Another limitation of this study was that the first questionnaire was completed before surgery, but this was not the baseline for orthodontic treatment. Patients already had orthodontic braces for a few months, which can influence the OHRQoL when they filled out the first questionnaire. Huang et al. compared surgery-first and orthodontic-first treatments. The orthodontic-first group experienced deterioration before surgery and suggested that pre-orthodontics could worsen the facial deformity [42]. Therefore, our last evaluation was 1–3 years after surgery. Not every patient had finished the orthodontic treatment. Choi et al. suggested that the best time for evaluating OHRQoL is 1 year after debonding [34].

Notably, we did not take into account a possible second operation that may have been required as a follow-up of the first surgery due to complications or a relapse. A second surgery could result in more discomfort and lower OHRQoL, influencing the answers to the questionnaire.

Another point that could influence the answers is that the consultation and surgeries were done by different oral maxillofacial surgeons of the Amsterdam UMC. This creates variation in preoperative preparations, provided information, manner of operation, and postoperative support.

Further long-term clinical studies should investigate the impact of orthognathic surgery on psychological well-being and OHRQoL in patients. This could lead to better preoperative and postoperative guidance for patients who undergo orthognathic surgery.

## Conclusion

The findings of the present study indicate that OHRQoL after orthognathic surgery in patients with various dentofacial deformities is reduced from baseline in the immediate postoperative period but improves over time. By 1 year, OHRQoL improves significantly after orthognathic surgery in patients with dentofacial deformities.
